# A Deep Neural Network for Gastric Cancer Prognosis Prediction Based on Biological Information Pathways

**DOI:** 10.1155/2022/2965166

**Published:** 2022-09-09

**Authors:** Jili Hu, Weiqiang Yu, Yuting Dai, Can Liu, Yongkang Wang, Qingfa Wu

**Affiliations:** ^1^School of Medical Informatics Engineering, Anhui University of Chinese Medicine, Hefei, Anhui, China; ^2^Anhui Computer Application Research Institute of Chinese Medicine, China Academy of Chinese Medical Sciences, Hefei, Anhui, China; ^3^The CAS Key Laboratory of Innate Immunity and Chronic Disease, University of Science and Technology of China, Hefei, Anhui, China; ^4^School of Life Sciences, University of Science and Technology of China, Hefei, Anhui, China; ^5^IBMC-BGI Center, The Cancer Hospital of the University of Chinese Academy of Sciences, Institute of Basic Medicine and Cancer (IBMC), Chinese Academy of Sciences, Hangzhou, Zhejiang 310022, China

## Abstract

**Background:**

Gastric cancer (GC) is one of the deadliest cancers in the world, with a 5-year overall survival rate of lower than 20% for patients with advanced GC. Genomic information is now frequently employed for precision cancer treatment due to the rapid advancements of high-throughput sequencing technologies. As a result, integrating multiomics data to construct predictive models for the GC patient prognosis is critical for tailored medical care.

**Results:**

In this study, we integrated multiomics data to design a biological pathway-based gastric cancer sparse deep neural network (GCS-Net) by modifying the P-NET model for long-term survival prediction of GC. The GCS-Net showed higher accuracy (accuracy = 0.844), area under the curve (AUC = 0.807), and F1 score (*F*1 = 0.913) than traditional machine learning models. Furthermore, the GCS-Net not only enables accurate patient survival prognosis but also provides model interpretability capabilities lacking in most traditional deep neural networks to describe the complex biological process of prognosis. The GCS-Net suggested the importance of genes (UBE2C, JAK2, RAD21, CEP250, NUP210, PTPN1, CDC27, NINL, NUP188, and PLK4) and biological pathways (Mitotic Anaphase, Resolution of Sister Chromatid Cohesion, and SUMO E3 ligases) to GC, which is consistent with the results revealed in biological- and medical-related studies of GC.

**Conclusion:**

The GCS-Net is an interpretable deep neural network built using biological pathway information whose structure represents a nonlinear hierarchical representation of genes and biological pathways. It can not only accurately predict the prognosis of GC patients but also suggest the importance of genes and biological pathways. The GCS-Net opens up new avenues for biological research and could be adapted for other cancer prediction and discovery activities as well.

## 1. Introduction

Gastric cancer (GC) is one of the deadliest tumors in the world and gastric adenocarcinoma (GAC) is the most common type of gastric cancer [1], with 95% of gastric malignancies being GAC [2]. Although early gastric cancer can be cured by surgical resection, the 5-year overall survival (OS) rate of advanced gastric cancer is less than 20% due to its easy recurrence and metastasis [4]. Therefore, it is imperative to improve the prognosis of gastric cancer patients, in order to guide personalized medical services and carry out tailored treatment plans.

Many types of genomic data have been acquired as a result of the advancements of next-generationhigh-throughput sequencing technology, including DNA methylation [5], mRNA [6], miRNA [6], and copy number variation (CNV) [7]. Because these datasets provide distinct viewpoints on cancer samples, combining multiomics datasets for cancer type prediction is advantageous. The Cancer Genome Atlas (TCGA) organization has released multiomics sequencing data for 33 cancer types [8], which is useful for comprehensive cancer analysis using multiomics data.

Deep learning (DL) algorithms have recently demonstrated remarkable performance in handling multiomics nonlinear data and numerous DL-based cancer multiomics analysis methods have been developed. Based on the combination of clinical and multiomics data, Tong suggested an integrative predictive model for colon cancer [9]. Using an autoencoder architecture, Chaudhary integrates multiomics data to predict hepatocellular carcinoma (HCC) survival. Hu developed a random forest deep feature selection (RDFS) and approach to increase gastric cancer prediction accuracy by combining the gene expression and copy number variation data [11]. Based on multiomics ensemble data, Xu employed a bidirectional deep neural network (BiDNN) model to predict the prognosis of gastric cancer [12]. Tufail summarizes DL models for cancer diagnosis and prognosis prediction tasks [13].

Although these models have revolutionized the diagnosis and predictions of cancers, they tend to be black boxes with poorly interpretable models. Conversely, machine learning models based on interpretable biomedical information may contribute to cancer genomic discovery and clinical prediction [14–16]. Hao et al. designed a pathway-associated sparse deep neural network (PASNet) to predict long-term survival in glioblastoma multiforme (GBM) accurately by incorporating biological pathways [17], but the hidden layers of the PASNet model are not entirely based on biological pathway information. Elmarakeby developed P-NET, a biologically informative deep learning model, to classify primary and castration-resistant prostate cancer (CRPC) [18], but the authors did not state why only 5 layers were chosen in the biological information pathway. These studies bring interpretability research to deep learning for cancer clinical prediction.

Using multiomics data to analyze the complex biological mechanisms of cancer patient survival is crucial; however, high-dimensional, nonlinear data pose computational challenges for survival analysis. In this study, we integrated multiomics data and designed a gastric cancer sparse deep neural network (GCS-Net) by modifying the P-NET model for gastric cancer prognosis, which can not only perform patient survival prognosis but also describe the complex biological process of prognosis. The GCS-Net is biologically interpretable with nodes in the neural network corresponding to biological genes and pathways, which can capture the nonlinear and hierarchical effects of biological genes and pathways on gastric cancer patient survival. Applying the GCS-Net to long-term survival prediction of GC, GCS-Net's accuracy, area under the curve (AUC), and F1 score are all higher than those of traditional machine learning models. Furthermore, genes and biological pathways discovered to be significant in the GCS-Net were validated as important genes and pathways for GC in previous biological and medical studies.

The remainder of the paper is organized as follows: Section 2 explains the datasets and data preprocessing procedure used in our study, the structure and operating principle of the GCS-Net, and the traditional machine learning models we compare the GCS-Net against in GC prognosis.  Section 3 compares the results of the GCS-Net with those of traditional machine learning models in GC prognosis and inspects the GCS-Net to uncover significant genes and biological pathways.  Section 4 presents a discussion of the results in Section 3. Finally, Section 5 provides the concluding remarks.

## 2. Materials and Methods

### 2.1. Datasets

We used the *R* tool “TCGA-assembler 2” [19] to download the GC dataset from TCGA (https://tcga-data.nci.nih.gov/tcga/). The dataset contains two types of multiomics data: copy number variation (CNV), somatic mutation, and clinical data. Integrating copy number alteration and somatic mutation data helps to reveal and predict survival time due to genomic variation in gastric cancer. The dataset has 295 samples, including 295 mutation data and 293 CNV data.

The GCS-Net network architecture is constructed based on the biological pathway database Reactome [20]. We download the Reactome pathway database from https://reactome.org/download-data, which contains three files: the gene matrix file ReactomePathways.gmt, the pathway name file ReactomePathways.txt, and the pathway parent-child relationship file ReactomePathwaysRelation.txt. From the parent-child relationship file, we create a hierarchical network with four levels of pathways, one layer of genes, and one layer of characteristics.

### 2.2. Data Preprocessing

Long-term survival (LTS) samples were those who lived for more than 60 months (independent of survival status), while short-term survival samples were those who died in less than 60 months (non-LTS). We obtained 183 non-LTS samples and 42 LTS samples, of which approximately 20% were LTS patients.

The CNV data were standardized to −2, −1, 0, 1, 2. CNV deletion was defined as −2 and CNV amplification as 2. Somatic mutation data were normalized to 1 and 0, with 1 denoting a gene with at least one site mutation and 0 denoting a gene with no mutation.

### 2.3. Construction of the Pathway Layers in the GCS-Net

**Figure 1:**
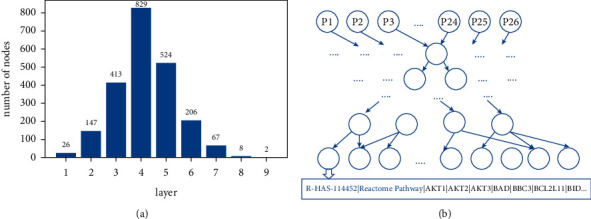
(a) The number of nodes in each layer of the network is constructed based on the Reactome pathway. The first layer has 26 nodes and the last layer has 2 nodes. (b). The parent-child relationship network layer constructed based on the Reactome pathway has a total of 9 layers, each node represents a pathway, and the node corresponds to the corresponding pathway gene.

We read the Reactome pathway file ReactomePathwaysRelation.txt, which contains the parent-child relationships in the pathway, and chose the human relationships by the keyword “HSA.” Then, we used the Python package NetworkX [21] to build a directed acyclic graph based on the chosen human relationships (Figure 1(b)). The distribution of the number of nodes in each layer is shown in Figure 1(a), in which the fourth layer has the largest number of nodes and the fifth layer ranks second.

To capture the relationship between gastric cancer information pathways and reduce network operations, we selected the first four layers to construct the pathway layers in the GCS-Net. In the directed acyclic graph, the directed edges point from parent pathways to child pathways they depend on, while in the GCS-Net, this is reversed, with the outputs of child pathway nodes serving as inputs of parent pathway nodes. Thus, the fourth layer of the directed acyclic graph is the first pathway layer in the GCS-Net, while the first layer of the directed acyclic graph is the last pathway layer in the GCS-Net.

### 2.4. The Architecture of the GCS-Net

**Figure 2:**
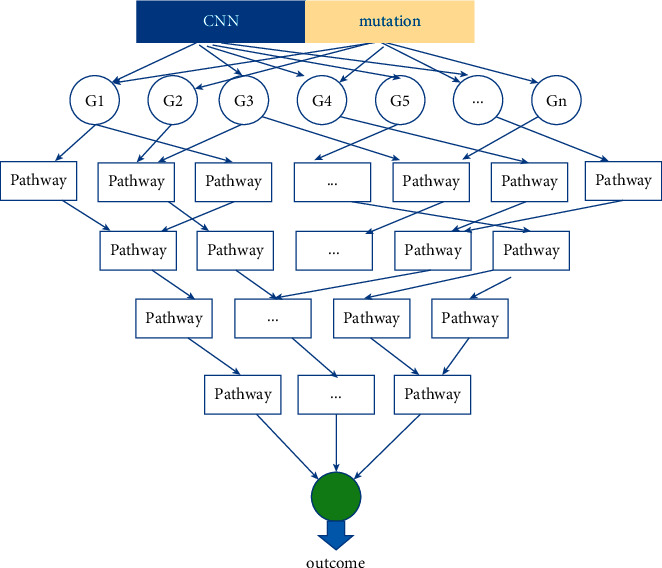
The architecture of the GCS-Net proposed to integrate multiomics data for the GC prognosis prediction. The structure of the GCS-Net consists of a feature layer (multiomics data), a layer of genes, and four layers of biological pathways based on Reactome, and the layers are directly sparsely connected.

As shown in Figure 2, one layer of feature data serving as an input layer, one layer of genes, and four layers of pathways make up the GCS-Net model. In this study, we use mutations and copy number variations as feature data, and we used the GCS-Net model with such multiomics data as the input to predict patient survival.

### 2.5. Operating Principle of the Gastric Cancer Sparse Deep Neural Network (GCS-Net)

Based on the Reactome-based network relationship built by NetworkX, we use TensorFlow's high-level API Keras to build multiple linear layers, with each layer followed by dropout and then an activation function.

The input layer represents feature data that need to be fed into the network for training, which is mutations and copy number variation data (encompassing copy number amplifications and copy number deletions) in this study. Each input node represents a feature and they are combined to form an m-column vector, denoted by *x*={*x*_1_, *x*_2_,…, *x*_*m*_}.

The gene layer consists of genes involved in the pathways of the first pathway layer. The connection between the input layer and the gene layer is established based on the fourth layer of the pathway database. Each node in the fourth layer of the pathway database is made up of a set of genes; thus, the connection between the input layer and the gene layer is a sparse connection, but not a full connection. We construct a binary adjacency matrix, *A* ∈ *P*^*n*×*m*^, where *n* is the number of pathways in the first pathway layer and *m* is the number of genes in the gene layer, to encode the connections between the gene layer and the first pathway layer. We set the value of the element *a*_*ij*_ of *A* to one if gene *j* belongs to some pathway *i*, and zero otherwise. This is a sparse coding model established based on the relationship between genes and pathways.

In the subsequent pathway layers, the connections between two adjacent pathway layers are determined by the pathway parent-child relationship in the Reactome pathway dataset and are stored in a binay mask matrix *M*, where *M* is a binary matrix created from parent-child relationships between the Reactome pathways. During the forward propagation calculation of the network, the output vector *y* of each layer is jointly determined by the input vector *x*, the weight matrix *W*, and the mask matrix *M*, forming a sparse network model. The calculation formula is as follows:

(1) $$\begin{array}{c} y=f\left(\left(W*M\right)*x+b\right), \end{array}$$

where *f* is the activation function. For each node, we use the following tanh activation function:

(2) $$\begin{array}{c} f=\tanh=\frac{\left(e^{2x}-1\right)}{\left(e^{2x}+1\right)}, \end{array}$$

and as a result, the value of each node remains in the range [−1, 1]. The activation function of the final output layer is the sigmoid function:

(3) $$\begin{array}{c} f=\frac{1}{\left(1+e^{-x}\right)}, \end{array}$$

which outputs a number in the range of (0, 1), with 0 representing good prognosis and 1 representing poor prognosis.

To measure the importance of each node in the network model, we use the DeepLIFT [22] gradient-based attribution method to rank the features in all layers. DeepLIFT utilizes a back-propagation method to propagate important signals from output neurons back through layers to the input [22]. The DeepLIFT scheme implemented in this study uses the GitHub library (https://github.com/kundajelab/deeplift).

In this work, to calculate the importance of nodes in each layer, each node needs to be assigned a score. Let *t* represents the target output and let *x*_1_, *x*_2_,…, *x*_*n*_ represent some intermediate layer neurons that are necessary to compute the target output. Let *t*^0^ denotes the reference activation of *t*.We define Δ*t* as the difference-from-reference:

(4) $$\begin{array}{c} \Delta t=t-t^{0}. \end{array}$$

DeepLIFT assigns contribution scores *C*_Δ*x*_*i*_Δ*t*_ to Δ*x*_*i*_ s.t.:

(5) $$\begin{array}{c} \sum_{i=1}^{n}C_{\Delta x_{i}\Delta t}=\Delta t, \end{array}$$

where *C*_Δ*x*_*i*_Δ*t*_ can be thought of as the amount of difference-from-reference in *t* that is attributable to the difference-from-reference of *x*_*i*_.

### 2.6. Parameters Optimization and Model Training

We split TCGA gastric cancer data set (containing somatic mutation and copy number data) into 80% training set, 10% validation set, and 10% test set for predicting survival. To make the model training converge smoothly, we initialize the learning rate to 0.001 and reduce it actively after every 100 epochs. The model is trained using the Adam optimizer [23]. We performed 1000 epochs of training and optimized parameters according to the cross entropy loss function:

(6) $$\begin{array}{c} L=\frac{1}{N}\sum_{i}-\left[y_{i}\cdot \;\log\left(p_{i}\right)+\left(1-y_{i}\right)\cdot \;\log\left(1-p_{i}\right)\right], \end{array}$$

where *N* represents the total number of samples, *y*_*i*_ is the label corresponding to sample *i*, and *p*_*i*_ is the LTS probability of sample *i* calculated according to the sigmoid function.

### 2.7. Methods for Comparison

In this work, we investigated the effectiveness of four traditional machine learning approaches in predicting the prognosis of stomach cancer (decision trees, support vector machines, logistic regression, and random forests). We utilized the scikit-learn package to implement these algorithms and used the default settings [24].

## 3. Results

### 3.1. Comparison of Weights between the GCS-Net Model and the Dense Network Model

There are much fewer weights in the GCS-NET sparse model than in a fully connected dense network with the same number of nodes. The number of weights of the sparse model is slightly higher than 83,347 (Table 1), while the fully connected dense network has more than 300 million weights. The formula for calculating the number of weights in a layer in the fully connected dense network is as follows:

(7) $$\begin{array}{c} w_{l}=n_{l}*\left(n_{l-1}+1\right), \end{array}$$

where *w*_*l*_ is the number of weights in a layer *l*,  *n*_*l*_ is the number of nodes in the same layer, and *n*_*l*−1_ is the number of weights in the previous layer. The formula for calculating the number of weights in a layer in the sparse network is as follows:

(8) $$\begin{array}{c} \text{weights}=M*W, \end{array}$$

where *M* is the mask matrix of each layer, with each element in *M* being 1 or 0 depending on whether or not the corresponding connection path with the parent-child relationship exists. *W* is the weight matrix of the layer.

### 3.2. Comparison with Other Methods

**Figure 3:**
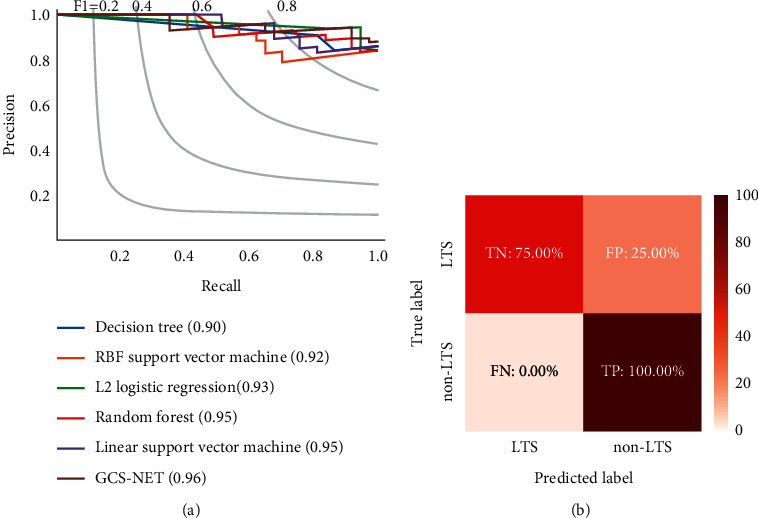
Prediction performance of the GCS-Net. (a) The AUPRC value of the GCS-Net outperforms other classical machine learning models on the test set. (b) The GCS-Net has a true negative rate (TN) of 75% and a true positive rate (TP) of 100% in the test set.

Traditional machine learning models such as decision trees, support vector machines, logistic regression, and random forests perform worse than the GCS-Net method. We trained the GCS-Net and these traditional machine learning models for long-term survival prediction of gastric cancer (GC), and the GCS-Net showed higher accuracy, area under the curve (AUC), and F1 score than previous traditional prediction classifiers (area under the receiver operating characteristic (ROC) curve (AUC) = 0.807, area under the precision-recall curve (AUPR) = 0.949, and accuracy = 0.844) (Table 2) (Figure 3(a)).

Evaluated on the test set, the GCS-Net model achieved a true negative rate of 75% (TN) and a true positive rate of 100% (TP), indicating that the model has a certain generalization and can classify samples that are not in the training set (Figure 3(b)).

### 3.3. Inspection and Interpretation of the GCS-Net

**Figure 4:**
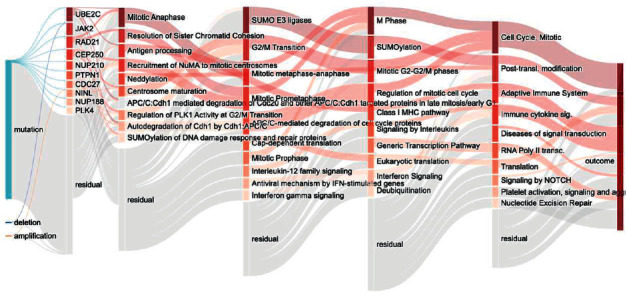
GCS-Net model pathway Sankey diagram. The Sankey diagram visualization shows the node importance and mutual drive of each layer of the GCS-Net model, and the nodes with darker colors are more important. The left most node represents the input feature data type; the nodes of the second layer represent the last layer of genes constructed according to the Reactome pathway; each subsequent layer represents a higher-level biological pathway; the last layer represents the prediction result.

To understand the connections and interactions between different mutations, copy number variations, genes, and biological pathways from input to output after training, we visualized the entire structure of the GCS-Net using a Sankey diagram (Figure 4).

From the figure, we can see that compared with copy number variation, mutation has a greater impact on the prognosis, which is consistent with the related studies of gastric cancer. To obtain the importance of each node, we use the DeepLIFT attribution method to calculate the node's contribution score to rank the nodes. UBE2C, JAK2, RAD21, NUP210, PTPN1, CDC27, NUP188, and PLK4 were the top-ranked genes, and they have been reported in related gastric cancer studies (Table 3).

At the same time, in the hidden layer of pathways, we found that mitotic anaphase, antigen processing, recruitment of NuMA to mitotic centrosomes, neddylation, centrosome maturation, SUMO E3 ligases, G2/M transition, M phase, SUMOylation, and cell cycle have an important impact on the prognosis of gastric cancer. These pathways involve cell cycle checkpoints, posttranslational modification, and transcriptional regulation. These pathways have been studied in the relevant gastric cancer prognostic literature (Table 4).

The expression level of the mitotic checkpoint BUB gene family is closely connected with tumor cell proliferation, according to the literature [33], and the BUB overexpression in gastric cancer is a proliferation-dependent phenomenon. Authored study on antigen processing and immune regulation in the response to tumors by Reeves and James [34]. Pan et al. discovered the SUMO E3 ligase CBX4 as a poor prognostic predictor in gastric cancer using a multipronged OMIC analysis [38].

## 4. Discussion of Results

Compared with traditional machine learning methods, the GCS-Net has better performance and significantly reduces the number of learning parameters. More importantly, it has an excellent model interpretability. Using the DeepLIFT method to measure the importance of different genes and pathways in predicting results, the GCS-Net found known genes related to gastric cancer, such as UBE2C, JAK2, RAD21, CEP250, NUP210, PTPN1, CDC27, NINL, NUP188, and PLK4. In addition, the GCS-Net also discovered important biological pathways, such as mitotic anaphase, resolution of sister chromatid cohesion, and SUMO E3 ligases. These important genes and pathways are documented in relevant gastric cancer biology literature.

Although our method has proved to be robust and reliable in predicting the prognosis of gastric cancer, there are still some concerns that need to be addressed. First, we found that the false-positive rate was high. One possible reason was the imbalance of samples in the dataset. Among them, there were only 42 samples with a good prognosis of gastric cancer with long-term survival greater than 5 years. Second, this experiment uses mutation data and copy number variation data in the multiomics data. If more omics data such as RNA and methylation data had been added, there might have been a higher prediction accuracy. Third, studies [42] have shown that clinical data also help to improve cancer prognosis prediction performance, which is a potential approach to improve model prediction performance.

## 5. Conclusions

Multiomics data analysis can be used to forecast cancer survival information. In this study, we developed the GCS-Net for predicting gastric cancer prognosis. The GCS-Net utilizes a biological pathway-based architecture and integrates multiomics data for prognosis prediction of gastric cancer.

In the future, we will add more omics data for prediction, use cross-validation to reduce the performance impact of low sample size, and collect more sample data for modeling. In addition, we will optimize the interpretability of deep neural networks through optimization algorithms, such as loss functions, to further improve the accuracy of the model. We will also consider applying this model to the prediction of gastric cancer types, such as diffuse and intestinal types [44].

Finally, the GCS-Net is a deep neural network with interpretable biological pathways for accurate gastric cancer prognosis. Neural networks based on biological information pathways offer a novel approach to biological discovery that might be used for a variety of additional cancer prediction and research applications. To more precisely assess the prognosis of gastric cancer patients, we will combine clinical data and multiomics data and analyze the effect of heterogeneity generated by diverse clinical characteristic data (including age, gender, and pathology) on the prognostic risk of gastric cancer patients.

## Figures and Tables

**Table 1:** GCS-NET network model parameters.

Layer (type)	Output shape	Param	Connected to
Inputs (InputLayer)	(none, 34380)	0	
h0 (Diagonal)	(none, 11460)	45840	Inputs[0][0]
dropout_0 (Dropout)	(none, 11460)	0	h0[0][0]
h1 (SparseTF)	(none, 1061)	22081	dropout_0[0][0]
dropout_1 (Dropout)	(none, 1061)	0	h1[0][0]
h2 (SparseTF)	(none, 447)	1512	dropout_1[0][0]
dropout_2 (Dropout)	(none, 447)	0	h2[0][0]
h3 (SparseTF)	(none, 147)	594	dropout_2[0][0]
dropout_3 (Dropout)	(none, 147)	0	h3[0][0]
h4 (SparseTF)	(none, 26)	174	dropout_3[0][0]
o_linear1 (Dense)	(none, 1)	11461	h0[0][0]
o_linear2 (Dense)	(none, 1)	1062	h1[0][0]
o_linear3 (Dense)	(none, 1)	448	h2[0][0]
o_linear4 (Dense)	(none, 1)	148	h3[0][0]
o_linear5 (Dense)	(none, 1)	27	h4[0][0]
Total		83347	

**Table 2:** The GCS-Net and other classic machine learning method model's scores.

Model	Accuracy	auc	aupr	f1	Precision	Recall
GCS-Net	0.844	0.807	0.949	0.913	0.840	1
L2 LogisticRegression	0.800	0.751	0.907	0.886	0.833	0.945
RBF support vector machine	0.733	0.628	0.916	0.846	0.804	0.891
Linear support vector machine	0.777	0.743	0.943	0.871	0.829	0.918
Random forest	0.800	0.785	0.946	0.886	0.833	0.945
Decision tree	0.755	0.692	0.893	0.857	0.825	0.891

**Table 3:** The top genes for survival prediction in GC by the GCS-Net.

Gene name	Reference
UBE2C	[25]
JAK2	[26]
RAD21	[27]
NUP210	[28]
PTPN1	[29]
CDC27	[30]
NUP188	[31]
PLK4	[32]

**Table 4:** The top pathway for survival prediction in GC by the GCS-Net.

Pathway name	Reference
Mitotic anaphase	[33]
Antigen processing	[34]
Recruitment of NuMA to mitotic centrosomes	[35]
Neddylation	[36]
Centrosome maturation	[37]
SUMO E3 ligases	[38]
G2/M transition	[39]
M phase	[40]
SUMOylation	[41]
Cell cycle	[42]

## Data Availability

Gastric cancer data are obtained from TCGA database (https://tcga-data.nci.nih.gov/tcga/). The Bioinformatics Pathway Database Reactome is from https://reactome.org/download-data.
